# Progressive Hand Stiffness and Numbness in a Child: An Atypical Neurological Presentation of Scheie Syndrome—A Case Report

**DOI:** 10.3390/neurolint17120205

**Published:** 2025-12-17

**Authors:** Ayidh Saad Alharthi, Chafik Ibrahim Hassan, Ali Alsayed Alsharkawy, Saeed Dhaifallah Saeed Alzahrani, Saif Ahmed Alzahrani

**Affiliations:** 1 Pediatric Neurology Unit, Department of Pediatrics, King Fahad Hospital, Al-Baha P.O. Box 204, Saudi Arabia; 2 Pediatric and Neonatology Department, King Fahad Hospital, Al-Baha P.O. Box 204, Saudi Arabiaazahrane2013@gmail.com (S.A.A.)

**Keywords:** Scheie syndrome, attenuated MPS I, mucopolysaccharidosis, hand stiffness, pediatric neurology

## Abstract

Background/Objectives: Scheie syndrome is the attenuated phenotype of mucopolysaccharidosis type I (MPS I), a lysosomal storage disorder resulting from partial deficiency of α-L-iduronidase. The attenuated clinical spectrum and absence of cognitive impairment often delay recognition. Early manifestations may mimic common pediatric conditions, leading to repeated evaluations without a definitive diagnosis. Methods: We describe a 12-year-old girl who presented with slowly progressive bilateral hand stiffness, weak grip strength, and intermittent sensory symptoms over one year. Her initial investigations—including laboratory studies, electrophysiology, imaging, and multispecialty evaluations—were unremarkable. Results: The gradual progression of symptoms involving joints, motor function, and vision prompted metabolic testing. Whole exome sequencing revealed a homozygous IDUA variant, and enzymatic testing confirmed markedly reduced α-L-iduronidase activity, establishing the diagnosis of Scheie syndrome. Early initiation of enzyme replacement therapy was pursued. Conclusions: This case emphasizes that children with unexplained musculoskeletal and sensory symptoms should be evaluated for attenuated MPS I, especially when routine studies are inconclusive. Heightened clinical suspicion can reduce diagnostic delay and improve long-term outcomes.

## 1. Introduction

Scheie syndrome represents the attenuated end of the mucopolysaccharidosis type I spectrum [1,2]. It often presents subtly, with clinical manifestations appearing gradually and sometimes remaining unnoticed for years. Children with attenuated MPS I typically have normal cognitive development [2,3], which can mislead clinicians into assuming a benign condition. Because the early symptoms are often nonspecific—such as mild joint stiffness, decreased flexibility, reduced endurance, or intermittent discomfort—the diagnostic process can become prolonged.

These children are commonly evaluated by multiple specialties, including orthopedics, neurology, rheumatology, ophthalmology, and cardiology. Despite multiple evaluations, the true metabolic cause may remain unrecognized. Limited awareness of attenuated MPS I further contributes to diagnostic delays [3,4]. For many clinicians, the absence of coarse facial features, organomegaly, or skeletal abnormalities reduces suspicion for a lysosomal storage disorder. However, the absence of these classic signs does not rule out attenuated MPS I.

Joint stiffness, particularly affecting the hands, may be the earliest and most persistent symptom [5,6]. Children may struggle with handwriting or fine motor tasks. They may have difficulty performing age-appropriate physical activities due to limited range of motion. These manifestations can easily be misinterpreted as mechanical joint issues or early rheumatologic disease. Normal inflammatory markers and unremarkable imaging may further delay recognition.

Vision changes, such as progressive refractive errors, may also emerge, but corneal clouding may be minimal or absent early in the disease. Cardiac involvement, particularly valvular abnormalities, tends to occur later in the clinical course [7]. The heterogeneity of symptoms highlights the need for clinicians to consider attenuated MPS I in children with unexplained, slowly progressive multisystem findings [3,7].

## 2. Case Presentation

The patient was a 12-year-old girl who presented with a one-year history of progressive bilateral hand stiffness. Her symptoms began subtly, with occasional difficulty writing and gripping objects. Over time, the stiffness became more noticeable, particularly in the morning, and she struggled with tasks requiring fine motor control. Her parents observed that she frequently dropped objects and avoided activities requiring prolonged hand use. The patient also reported intermittent numbness affecting the palmar surfaces of both hands, which fluctuated in intensity. Despite these symptoms, her daily functioning remained relatively preserved early in the illness.

**Figure 1:**
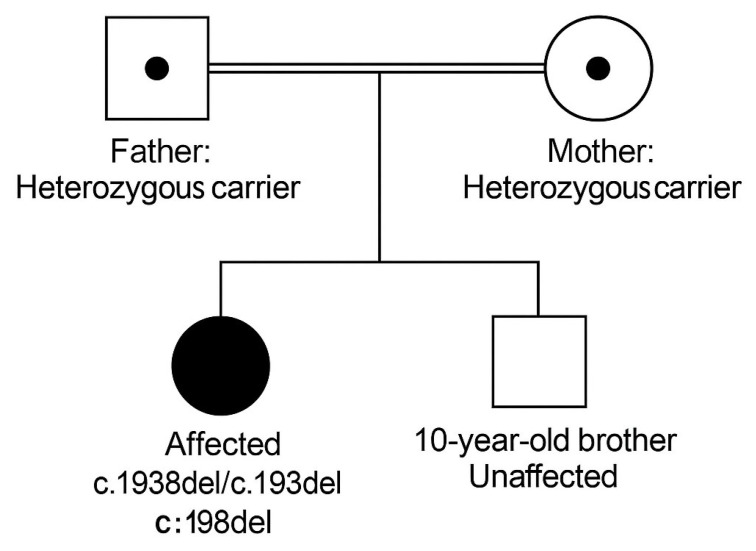
Family Pedigree of the Proband. The proband is homozygous for the IDUA c.1938del (p.Pro648Hisfs*) variant. Mother: Heterozygous carrier. Father: Heterozygous carrier. Sibling (10-year-old boy): Genetically unaffected. Parents are first cousins.

Her medical history was unremarkable, with normal developmental milestones and no previous neurological or rheumatologic concerns. Her parents, who are consanguineous (see Figure 1 for the family pedigree), reported that a maternal aunt had experienced similar hand symptoms but was never formally evaluated.

Neurological examination revealed reduced grip strength and decreased pinprick sensation in both palms. On inspection, both hands showed reduced active and passive range of motion, particularly at the metacarpophalangeal and proximal interphalangeal joints. The fingers were held in a semi-flexed posture with noticeable limitation in extension. There was no swelling, erythema, or joint warmth, and the palmar skin appeared smooth and slightly taut. These findings were consistent with her reported functional difficulties. Muscle bulk and tone were otherwise normal, and reflexes were symmetrical. Systemic examination revealed no dysmorphic features, no hepatosplenomegaly, and no musculoskeletal deformities.

Laboratory evaluations, including inflammatory markers, thyroid function, vitamin levels, and autoimmune serologies were within normal limits. Nerve conduction studies demonstrated normal motor and sensory responses, ruling out peripheral neuropathy or entrapment syndromes. Radiographs showed no evidence of dysostosis multiplex or joint degeneration. Ophthalmologic evaluation identified hyperopia but no corneal clouding. The combination of progressive joint stiffness, sensory symptoms, normal basic investigations, and consanguinity raised suspicion for a metabolic disorder.

Whole exome sequencing identified a homozygous IDUA variant, and enzymatic testing confirmed significantly reduced α-L-iduronidase activity. Key diagnostic findings are summarized in Table 1.

The patient was diagnosed with Scheie syndrome and initiated on enzyme replacement therapy. Her follow-up plan included neurology, cardiology, ophthalmology, physiotherapy, and genetic counseling.

## 3. Discussion

This case underscores the diagnostic challenges of identifying attenuated MPS I in pediatric patients [3]. The subtle nature of early symptoms, combined with normal routine investigations, can easily mislead clinicians. Joint stiffness and reduced range of motion are hallmark features [5,6], yet they are frequently attributed to benign causes. Nerve conduction studies may be normal despite clinically significant symptoms because early tissue infiltration affects tendons and connective tissue rather than nerves directly [7,8]. The absence of corneal clouding, coarse facial features, or skeletal abnormalities further reduces suspicion [3]. Genetic testing and enzymatic assays are essential for confirming the diagnosis [8], especially when conventional screening tests are unrevealing.

From a pathophysiological perspective, the accumulation of glycosaminoglycans in Scheie syndrome primarily affects connective tissues, tendons, joint capsules, cardiac valves, and ocular structures [1,2]. Because α-L-iduronidase activity is only partially deficient in the attenuated form, the rate of GAG deposition is much slower compared to severe MPS I [9]. This slow progression often prevents early development of overt dysmorphism or organomegaly, contributing to the diagnostic challenge [4]. Neurological manifestations in attenuated MPS I are generally peripheral and mechanical rather than central, arising from infiltration of periarticular tissues, tendon thickening, or compression of small peripheral nerves [7,8]. These mechanisms may produce stiffness, limited range of motion, and sensory disturbances, yet electrophysiological studies may remain normal until later stages because the pathology is primarily extraneural in early disease.

Clinically, the patient’s presentation mimicked several more common conditions encountered in pediatric practice. Mild morning stiffness, intermittent numbness, and reduced grip strength initially suggested early rheumatologic disease or overuse injury [5]. The absence of joint swelling, normal inflammatory markers, and unremarkable imaging studies reasonably directed clinicians away from inflammatory arthritis [6]. Similarly, the sensory symptoms raised suspicion for entrapment neuropathies such as early carpal tunnel syndrome, yet electrophysiology did not support this [7]. Because the symptoms evolved gradually, it was easy to attribute them to benign musculoskeletal issues rather than a systemic lysosomal storage disorder [3]. This reinforces how attenuated MPS I can blend into the background of general pediatric complaints, delaying metabolic investigation.

The differential diagnosis in such cases is often broad and includes juvenile idiopathic arthritis, early connective tissue disease, hereditary neuropathies, repetitive strain injuries, and functional musculoskeletal pain (Table 2) [5,6,7].

When these possibilities are excluded through thorough evaluation, clinicians should maintain awareness of less common systemic causes of progressive joint dysfunction. Pediatric neurologists may be among the first specialists to evaluate these children because sensory symptoms and fine motor difficulties are often the earliest manifestations noticed by families [8]. For this reason, neurologists play a critical role in recognizing patterns suggestive of an underlying storage disorder—such as symmetric involvement, gradual progression, preserved strength, normal inflammatory markers, and multisystem involvement that may be easily overlooked [3].

The multisystem nature of attenuated MPS I also provides important diagnostic clues. Although our patient did not exhibit classic corneal clouding, she did experience progressive refractive error, an early ocular manifestation reported in attenuated phenotypes [2,3]. Likewise, although her cardiac evaluation was normal at the time of diagnosis, valvular disease is common in Scheie syndrome and may appear later in childhood or adolescence [10,11]. Subtle limitations in flexibility, decreased endurance, and restricted joint mobility may also precede more characteristic musculoskeletal findings such as tendon thickening or contractures [4,6]. Recognizing these early signs can significantly shorten diagnostic delays.

Genetic testing and enzymatic assay remain indispensable for confirming the diagnosis. Whole exome sequencing allows clinicians to identify pathogenic or potentially pathogenic variants in genes associated with rare diseases. However, because attenuated MPS I results from a spectrum of IDUA variants with variable phenotypic consequences, enzymatic confirmation is essential [8,9]. In this case, markedly reduced α-L-iduronidase activity provided definitive evidence supporting the genetic findings [12]. This combined approach is particularly valuable in clinical settings where urinary GAG analysis is unavailable or unreliable.

Early diagnosis is vital not only for initiating disease-modifying therapy but also for preventing irreversible complications. Enzyme replacement therapy with laronidase has demonstrated meaningful improvements in joint mobility, endurance, pulmonary function, and overall quality of life in attenuated MPS I [12]. Although ERT does not cross the blood–brain barrier and is limited in addressing CNS pathology, cognition is typically preserved in Scheie syndrome, making ERT an effective therapeutic option. Earlier initiation increases the likelihood of slowing disease progression and preserving motor function [11]. Alongside ERT, regular monitoring with cardiology, ophthalmology, orthopedics, pulmonary care, and physiotherapy is essential to address systemic involvement and optimize long-term care [6,10,11].

Another important aspect to consider in attenuated MPS I is the variability in clinical expression, even among individuals carrying the same genetic variant. This phenotypic diversity highlights the complexity of genotype–phenotype correlations in Scheie syndrome and reinforces the need for individualized assessment [4,9]. Some patients may demonstrate early joint involvement and preserved vision, while others may show early ocular abnormalities or mild cardiac findings before musculoskeletal symptoms become prominent. Such variability contributes to diagnostic difficulty and may delay recognition, especially when symptoms do not follow classic patterns [3]. Additionally, the slow disease progression may cause families and clinicians to underestimate symptoms or attribute them to lifestyle factors or benign developmental differences. Understanding this variability encourages clinicians to adopt a broader diagnostic perspective when encountering persistent, unexplained, multisystem symptoms in children. Recognizing the possibility of attenuated MPS I earlier in the disease course may facilitate timely therapeutic intervention and improve long-term functional outcomes.

Overall, this case highlights the need for increased awareness of attenuated MPS I and underscores how early recognition can dramatically alter a patient’s clinical trajectory. A heightened index of suspicion, combined with careful clinical observation and appropriate use of metabolic testing, remains key to ensuring timely diagnosis and optimizing outcomes for children with Scheie syndrome.

In addition to these clinical considerations, it is important to acknowledge that delayed diagnosis in attenuated MPS I is a well-documented global issue [3,4]. Many children undergo years of evaluations before the underlying metabolic cause is identified, especially when early symptoms appear mild or fluctuate over time. Increasing clinician awareness, improving access to enzymatic testing, and promoting early use of genetic panels may help shorten this delay [1,2]. Earlier recognition ultimately allows children to receive appropriate therapy before irreversible tissue damage occurs [12].

In attenuated MPS I, the diagnostic challenge is magnified by the subtle and slowly progressive nature of early symptoms. Many children initially present to orthopedic or neurology clinics with isolated hand complaints, often prompting evaluations focused on mechanical or peripheral nerve conditions. However, studies have shown that tendon infiltration and periarticular glycosaminoglycan deposition occur years before overt skeletal abnormalities become visible on radiographs [4,6]. This explains why early imaging and electrophysiology may appear normal despite functional impairment [7,8]. The dissociation between symptoms and diagnostic findings often misleads clinicians and contributes to substantial delays in recognition.

A key differentiating feature in attenuated MPS I is the pattern of progressive joint limitation without inflammation. While rheumatologic conditions such as juvenile idiopathic arthritis typically present with pain, swelling, and elevated inflammatory markers, children with Scheie syndrome often have painless, symmetrical stiffness with preserved joint architecture. This mechanical pattern results from gradual thickening of tendons and joint capsules rather than synovial inflammation. Recognizing this clinical distinction is essential for avoiding prolonged misclassification as rheumatologic disease [1,2,3,8].

Comparatively, more severe MPS I phenotypes—such as Hurler and Hurler–Scheie syndromes—present much earlier in life with coarse facial features, developmental delay, organomegaly, and characteristic skeletal changes. In attenuated forms, however, cognitive development is preserved and dysmorphic features may be minimal or absent. This phenotypic variability reflects residual enzyme activity and highlights the necessity of considering attenuated MPS I even in children who appear developmentally normal. Moreover, physical findings such as reduced endurance, decreased flexibility, and progressive refractive errors may provide important early clues to systemic involvement.

The role of diagnostic imaging in attenuated MPS I continues to evolve. While conventional radiographs may remain normal for years, advanced imaging techniques—including musculoskeletal ultrasound and MRI—have demonstrated greater sensitivity in detecting tendon thickening and periarticular tissue changes. Incorporating these modalities earlier in the diagnostic process may improve detection in children with unexplained joint stiffness. Similarly, urinary glycosaminoglycan levels, though useful, may be normal or only mildly elevated in attenuated phenotypes, further reinforcing the importance of enzymatic and genetic testing as definitive diagnostic tools.

The global literature consistently reports prolonged diagnostic delays in attenuated MPS I, often exceeding 5–7 years from symptom onset. These delays result not only from subtle clinical manifestations but also from limited awareness among clinicians. Early recognition has significant implications for disease progression, as enzyme replacement therapy is most effective before irreversible tissue changes occur. In addition to improving joint mobility and endurance, timely therapy can slow the development of cardiac valve disease and reduce long-term functional impairment. This case reinforces the importance of maintaining a broad differential diagnosis and integrating metabolic testing into the evaluation of persistent, unexplained musculoskeletal and sensory symptoms in children.

## 4. Learning Points

- Attenuated MPS I should be considered in children with unexplained, progressive hand stiffness, fine motor difficulties, or sensory disturbances despite normal routine investigations.
- Normal radiographs, electrophysiology, and inflammatory markers do not exclude lysosomal storage disorders. Consanguinity and early multisystem involvement (vision, joints, endurance) are key diagnostic clues.
- Early use of metabolic testing—including enzymatic assays and WES—can prevent years of diagnostic delay.

## 5. Pathophysiology Insights

Attenuated MPS I results from partial deficiency of α-L-iduronidase, leading to slow accumulation of dermatan and heparan sulfate in connective tissues. Early deposition affects tendons, joint capsules, and ocular tissues, producing mechanical symptoms rather than neurologic deficits. This explains why electrophysiology may remain normal early in the disease. Progressive GAG accumulation later results in reduced joint mobility, tendon thickening, and impaired fine motor function. Ocular involvement may begin with refractive errors before overt corneal clouding appears.

## 6. Global Perspective on Delayed Diagnosis

Delayed diagnosis of attenuated MPS I is a well-recognized global problem. Children frequently undergo years of evaluations for rheumatologic, orthopedic, or neurologic disorders before metabolic testing is considered. Studies report diagnostic delays ranging from 3 to 10 years, driven by early nonspecific symptoms and normal routine investigations. Increased clinician awareness and wider use of enzymatic assays and genetic testing have been shown to significantly reduce delays.

## 7. Conclusions

Early diagnosis of Scheie syndrome requires awareness of subtle clinical signs and persistence in evaluating unexplained multisystem symptoms. Children with progressive hand stiffness and sensory changes, especially with normal investigations, should be evaluated for attenuated MPS I. Genetic and enzymatic testing remain essential for diagnosis, and early enzyme replacement therapy can improve long-term outcomes. A multidisciplinary approach is crucial for comprehensive care. Early identification not only improves functional outcomes but also prevents irreversible complications that may develop if the disorder remains undiagnosed. Clinicians must maintain a high index of suspicion when evaluating children with persistent, unexplained joint limitations, sensory abnormalities, or slowly progressive symptoms that span more than one organ system. This case highlights how careful clinical observation, combined with consideration of rare metabolic diagnoses, can significantly change the patient’s disease trajectory and quality of life.

## Figures and Tables

**Table 1:** Summary of Diagnostic Findings.

Category	Findings
Neurological Examination	Reduced grip strength, decreased pinprick sensation, normal tone, normal reflexes.
Systemic Examination	No dysmorphic features, no organomegaly.
Laboratory Tests	Normal CBC, ESR, CRP, thyroid panel.
Electrophysiology	Normal nerve conduction study; no neuropathy.
Radiographs	Normal skeletal structure; no dysostosis multiplex.
Ophthalmology	Hyperopia; no corneal clouding.
Genetic Analysis	Homozygous IDUA variant: NM_000203.3:c.1938del (p.Pro648Hisfs*)—frameshift, VUS with strong evidence; consistent with attenuated MPS I.
Enzyme Assay	α-L-iduronidase activity markedly reduced: 0.7 μmol/L/h (Reference > 1.5 μmol/L/h); enzymology performed on dried blood spot.

**Table 2:** Differential Diagnoses.

Category	Findings
Juvenile Idiopathic Arthritis	Inflammatory markers elevated; joint swelling; pain.
Carpal Tunnel Syndrome	Median nerve symptoms; abnormal electrophysiology.
Hereditary Neuropathies	Progressive distal weakness; abnormal NCS.
Mechanical/Overuse Injury	Activity-related; non-progressive.
Connective Tissue Disease	Autoantibodies positive; systemic involvement.
Other Lysosomal Storage Disorders	Variable; confirmed by enzyme/genetic testing.

## Data Availability

No new data were created or analyzed in this study.
